# In vitro and ex vivo modeling of enteric bacterial infections

**DOI:** 10.1080/19490976.2022.2158034

**Published:** 2022-12-28

**Authors:** Nayere Taebnia, Ute Römling, Volker M. Lauschke

**Affiliations:** a Department of Physiology and Pharmacology, Karolinska Institutet, Stockholm, Sweden; b Department of Microbiology, Tumor and Cell Biology, Karolinska Institutet, Stockholm, Sweden; c Dr Margarete Fischer-Bosch Institute of Clinical Pharmacology, Stuttgart, Germany; d University of Tübingen, Tübingen, Germany

**Keywords:** Organotypic culture, microphysiological systems, infection models, intestine, monolayer cell culture, monolayer cell culture

## Abstract

Enteric bacterial infections contribute substantially to global disease burden and mortality, particularly in the developing world. *In vitro* 2D monolayer cultures have provided critical insights into the fundamental virulence mechanisms of a multitude of pathogens, including *Salmonella enterica* serovars Typhimurium and Typhi, *Vibrio cholerae, Shigella* spp., *Escherichia coli* and *Campylobacter jejuni*, which have led to the identification of novel targets for antimicrobial therapy and vaccines. In recent years, the arsenal of experimental systems to study intestinal infections has been expanded by a multitude of more complex models, which have allowed to evaluate the effects of additional physiological and biological parameters on infectivity. Organoids recapitulate the cellular complexity of the human intestinal epithelium while 3D bioengineered scaffolds and microphysiological devices allow to emulate oxygen gradients, flow and peristalsis, as well as the formation and maintenance of stable and physiologically relevant microbial diversity. Additionally, advancements in *ex vivo* cultures and intravital imaging have opened new possibilities to study the effects of enteric pathogens on fluid secretion, barrier integrity and immune cell surveillance in the intact intestine. This review aims to present a balanced and updated overview of current intestinal *in vitro* and *ex vivo* methods for modeling of enteric bacterial infections. We conclude that the different paradigms are complements rather than replacements and their combined use promises to further our understanding of host-microbe interactions and their impacts on intestinal health.

## 1. Introduction

The intestine of humans and animals is a fascinating organ characterized by an intricate mutual interplay between the host and the commensal microbiome that covers approximately 200 m^2^, making it the largest organ surface area of the human body. The vast majority of intestinal microbes are autochthonous and beneficial for human health whereas others can cause intestinal injury or disease via an arsenal of specific virulence factors that include the production of toxins, toxic metabolites or the invasion of intestinal epithelial cells.^1,2^ The gastrointestinal microbiome plays a predominant role in colonization resistance throughout the animal kingdom.^3,4^ Tightly controlled interactions between the host and the microbiome are thus essential to prevent dysbiosis and maintain intestinal health.^5^ However, a wide variety of Gram-negative and Gram-positive pathogens from diverse branches of the phylogenetic tree can cause infectious disease along the gastrointestinal tract. Besides acute infections, long-term subclinical dysbiosis in combination with environmental, nutritional and genetic factors can initiate a wide variety of inflammatory and autoimmune diseases, metabolic disorders, but also neuropsychiatric diseases and cancers.^6–9^ Furthermore, reduction of microbiome diversity through excessive use of antibiotics or impairment of its regenerative capacity, e.g. by appendectomy, can lead to imbalances in inter-microbe and microbiome-host crosstalk.

The intestinal barrier separates the gut lumen, the outside environment, from underlying tissues and controls the uptake of nutrients and xenobiotics, while restricting invasion of pathogens. The innermost layer of the intestinal mucosa is the epithelium, a lining consisting of a single layer of cells that are tightly connected by different cell junction complexes to provide an efficient barrier.^10^ This epithelial layer is primarily composed of enterocytes, but also includes mucin-secreting goblet cells, enteroendocrine cells, as well as various specialized cell types involved in antimicrobial defense, such as Paneth cells, which secrete antimicrobial factors, M cells, which sample and transport antigens to the underlying lymphoid tissue, as well as chemosensory tuft cells that protect against parasitic infections.^11^ The epithelial cell lining is apically covered by mucin layers, which are differentially functionalized with antimicrobial tools and protect epithelial integrity.^12,13^ The basolateral side in the lamina propria is speckled by resting immune cells. Microbiome-immune cell crosstalk promotes immune homeostasis, for instance by facilitating the extrathymic development of CD4^+^ regulatory T cells with immunosuppressive activity.^14^

Pathogens can traverse these barriers via paracellular or transcellular pathways. Paracellular crossing of the intestinal lining constitutes a process in which bacteria breach the tight connections between enterocytes. Paracellular permeability is increased upon inflammation, and, once integrity is compromised, leads to the infiltration of pathogens into the underlying submucosal layers or into the circulation.^15,16^ Alternatively, pathogens can infiltrate transcellularly via apical invasion of enterocytes followed by basolateral exocytosis. Both autochthonous and invading microbes can affect mucin production and alter the composition of the mucus layers with important consequences for epithelial maturation, barrier function and immune cell activation.^17,18^

Emulating the human intestine *in vitro* is of importance for mechanistic investigations into enteric infections, as well as for drug discovery programs focused on gastrointestinal diseases. For more than five decades, 2D monolayer cultures of human immortalized cell lines have served as the main *in vitro* paradigm to study infectious disease biology. While reductionistic, those systems have provided fundamental insights into the virulence and pathogenesis of various enteric bacterial infections and remain valuable tools for high throughput investigations that unravel molecular mechanisms of infection processes with unprecedented detail. In recent years, these models were complemented with a variety of bioengineered intestinal *in vitro* and *ex vivo* systems, including organoid cultures, scaffold-based 3D models and microfluidic systems. These models allow to capture intestinal phenotypes at the cellular and molecular level more accurately. In addition, they enable the investigation of effects related to mucus production and composition, symbiont-pathogen interactions, immune crosstalk and intestinal peristalsis – features that are not possible to study in conventional 2D *in vitro* models. However, these advantages come at the expense of increased complexity and reduced throughput. In this review, we provide an updated overview of *in vitro* and *ex vivo* models of enteric bacterial infections and discuss key examples where these different systems have advanced our understanding of the pathogenesis of infections with *Salmonella enterica* serovars Typhimurium and Typhi, *Vibrio cholerae*, *Shigella* spp., *Escherichia coli* and *Campylobacter jejuni*. For other intestinal pathogens we refer the interested reader to informative recent reviews.^19–22^ We furthermore highlight current frontiers where emerging complex intestinal model systems can provide added value.

## 2. Intestinal monolayer culture models

Historically, 2D cultures of human immortalized cell lines have served as the first *in vitro* systems to study infectious disease.^23^ In this approach, cells are cultured as monolayers on flat stiff surfaces, e.g. in petri dishes, tissue culture flasks or multi-well plates. For facultative intracellular pathogens, application of the aminoglycoside antibiotic gentamicin, which does not penetrate host cells and thus exclusively inhibits extracellular bacteria, allows to distinguish between intra- and extracellular replication. This gentamicin protection assay constitutes a classical method for quantifying pathogen invasiveness and intracellular replication that has led to the identification of multiple bacterial virulence factors by comparing wild type and mutant bacterial cells.^24^ While monolayer culture formats are highly scalable, inexpensive and amenable to high-throughput analyses, the phenotype of these cells is not fully differentiated. Furthermore, these models do not allow easy distinction between events occurring at the apical or basolateral side, rendering them unsuitable for studies involving cell polarity. To ameliorate these limitations, transwell cultures have been developed in which monolayers are cultured on nano porous membranes that separate two compartments.^25^ Transwell culture promotes the *in vitro* development of a polarized intestinal epithelium and these systems are extensively used for investigations of intestinal permeability and polarity.^26^ However, both flat culture dishes and transwells are mostly made from polystyrene or other thermoplastics, which are not oxygen permeable, have unfavorable mechanical properties and are prone to drug absorption of hydrophobic molecules.^27,28^

The most popular cell models for the study of enteric infectious disease biology are undifferentiated cell lines, such as the cervical epithelial cell line HeLa, Henle-407 and the kidney epithelial cell line HEK293, as well as the enterocyte-derived colorectal adenocarcinoma cell lines Caco-2 and HT29. Upon reaching confluency, Caco-2 cells exhibit features such as tight junction-mediated barrier function, apical brush border formation and elevated expression of intestinal enzymes, receptors and transporters.^29^ Caco-2 cells can furthermore be induced to transdifferentiate into M cells by co-culture with Raji B cells, a human B lymphoblastoid cell line ^30^ while HT29 cells and particularly the HT29-MTX subclone, can differentiate into mucus-producing goblet cells. Immortalized macrophage-like cell lines such as murine RAW264.7 and human J774.1 and THP-1 are frequently applied to assess interactions between enteric pathogens and basolaterally recruited immune cells that also cross the epithelial barrier to target pathogens in the lumen.

The advantages of immortalized cell lines are that they provide continuous, inexpensive and readily available supply of isogenic cellular material. Overall, however, cell lines are highly dedifferentiated, as evidenced by reduced or absent expression of multiple enterocyte-specific genes, including *SLC5A1, MTTP, VLDLR, PXR* (*NR1I2*) and *MUC4*.^31,32^ Immortalized cell lines also do not mimic the expression and diversity of *in vivo* toll-like receptor expression, which can impact the observed responses to microbial stimuli.^33^ Furthermore, they exhibit significantly lower levels of inflammasome activation and pyroptotic cell death, resulting in impaired extrusion of infected cells from polarized epithelia and, eventually, higher pathogen loads.^34,35^

As an alternative to epithelial cell lines, primary intestinal cells or dissociated intestinal organoids can be used, while bone marrow-derived macrophages (BMDM) provide an alternative to immortalized immune cells. The molecular signatures of these cells more closely resemble their *in vivo* counterparts and allow to investigate effects of inter-individual differences.^36,37^ However, they are more difficult to acquire, limited in material and the cells lose their phenotypic resemblance after a few passages.

**Figure 1.**
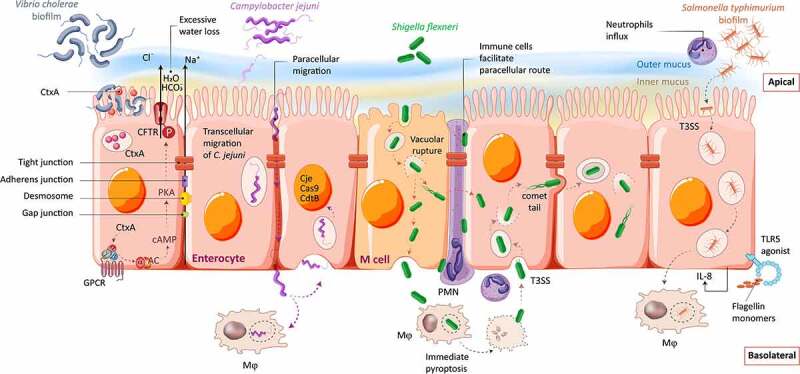
**Overview of key infectious mechanisms of enteric bacteria revealed using 2D monolayer cultures**. Illustration showing the mechanism of invasion and bacteria-host interactions for *Vibrio cholerae, Campylobacter jejuni, Shigella flexneri* and *Salmonella* Typhimurium. AC = adenylate cyclase; CdtB = virulence factor with DNase I activity; CjeCas9 = virulence factor causing unspecific DNA damage; CtxA = cholera toxin A; GPCR = G-protein coupled receptor; Mφ = macrophage; PMN = polymorphonuclear leukocyte; T3SS = type III secretion system.

A main advantage of monolayers is the standardized experimental setup, reproducibility, and applicability for drug studies. They have been successfully utilized to pioneer the delineation of different mechanisms of infection employed by a variety of bacterial enteropathogens and their toxins (Figure 1). However, most monolayer cultures comprise only enterocytes with only few studies attempting to integrate also other intestinal cell types, such as goblet cells and immune cells.^38–40^ Additionally, the vast majority of monolayer cultures are conducted under static conditions, which results in rapid bacterial overgrowth, thus strongly limiting culture times and impairing the investigation of stable host-microbiome interactions.

## 3. Modeling of enteric bacterial infections in monolayer cultures

Reductionistic monolayer cultures can aid in the characterization and dissection of the molecular events occurring upon infection. However, despite their relatively low complexity, multiple experimental parameters remain important to consider. Infectivity of pathogens can be strongly affected by different growth conditions prior to infection. One example of this phenomenon is displayed by *S*. Typhimurium, which is less prone to invade epithelial cell lines when grown on an agar plate where it forms multicellular aggregates and does not express its major virulence factor, the type III secretion system-1 (T3SS-1), whereas it is substantially more invasive when grown in liquid culture.^41^

Media composition, CO_2_ levels and nutrient supply also impact a multitude of molecular events, including signal transduction by bacterial two-component signaling systems based on the phosphotransfer from a histidine protein kinase to a response regulator, second messenger cyclic di-GMP and cyclic AMP signaling networks and chemotactic signaling. For instance, culture conditions that differ by ionic strength and the presence of digestive enzymes thereby mimicking the ileal or colonic environment, were sufficient to rewire metabolic pathways and production of virulence factors including Shiga toxin of enterohemorrhagic *E. coli*.^42^ In addition to differences in virulence factor expression, environmental conditions and nutrient supply can determine the extent of immunostimulation by microbial associated molecular patterns,^43^ such as regulation of flagellin secretion by cyclic di-GMP,^44^ as well as the decision to adhere to epithelial cells and form biofilms, all of which affect disease outcome.^45,46^

Infectivity is majorly modulated by the commensal microbiome and, thus, interaction with non-pathogenic microbes is also important to consider *in vitro*. Colonization resistance of the residing microbiota is reliant on various mechanisms such as production of colicins, inhibition of pathogens by metabolic products and the type VI secretion system (T6SS).^47–49^ A major protective mechanism of the microbial flora against pathogens is nutritional competition. *In vitro* studies found that commensal *E. coli* metabolize mono- and disaccharides available in the mucus, which was subsequently demonstrated to determine colonization success. Provision of mucus sugars to a commensal and an *E. coli* O157:H7 strain, which competes for the mucus niche, showed that the pathogenic strain used a distinct saccharide pool, which might provide a competitive colonization advantage.^50^ The T6SS, a needle-like nano-machine injecting effector proteins into target microbes and phagocytic cells facilitates virulence by altering cellular motility,^51^ culling bacterial competition ^52^ and killing host macrophages.^53^ Mucus activates the T6SS of *Vibrio cholerae*, which, in turn, is counteracted by the resident microbiota via modification of bile acids, which exhibit inhibitory functions.^54^ Notably, the T6SS can be used by both pathogenic and commensal bacterial to compete for enteric niches.^55^

Inter-experimental variability can moreover be founded in genetic differences of the utilized bacterial strains. For instance, even a single nucleotide polymorphism can alter fitness and interaction with host cells. One example is a missense mutation found in the cache 1 signaling domain of the di-guanylate cyclase STM1987 of the invasive *Salmonella enterica* serovar Typhimurium ST131 clone that recently emerged in sub-Saharan Africa.^56^ This variant was shown to drastically reduce cellulose production and displayed increased fitness both *in vitro* and *in vivo* virulence models.

Combined, these examples illustrate that, in addition to the selection of the cell model and culture system, genetic differences between bacterial strains, nutrient and media compositions as well as interactions with commensal bacteria constitute important factors that need to be considered for investigations of enteric infections. In the following, we highlight key insights into the pathobiology of enteric bacterial infections resulting from *in vitro* studies in monolayer cultures.

### 3.1 *Shigella* spp

The *Escherichia coli* subspecies *Shigella* spp., evolved multiple times through significant genome alterations and the acquisition of a large virulence plasmid. *Shigella* spp. causes shigellosis, a bacillary dysentery characterized by invasion of the epithelial lining with subsequent transcellular spreading. *Shigella* spp. infection is hallmarked by the uptake of bacteria into M cells, transcytosis, and subsequent macrophage uptake, followed by rapid escape via pyroptosis and basolateral invasion of enterocytes. In addition, using high-resolution microscopy of Caco-2/Raji B transwell cultures infected with *S. flexneri*, it was recently demonstrated that the bacteria can also directly spread from M cells to neighboring enterocytes, thereby evading adaptive immunity.^57^ Once in enterocytes, *Shigella* hijacks the host actin polymerization machinery leading to high intracellular motility and inflammation-mediated disruption of the intestinal epithelium. Initially, Shiga toxin produced by *Shigella dysenteriae* 1 was assumed to be the reason for the disease symptoms. Then, in a series of seminal experiments, the first published in 1964, cellular invasion of the HeLa cell line by the virulent wild type *S. flexneri* 2a, but not by its avirulent colony variant has been identified as a key virulence property.^58^ Subsequent early *in vitro* cell line experiments conducted most likely with spontaneous mutant strains of *S. dysenteriae* 1 moreover allowed to dissect the contributions of different virulence traits, such as host cell invasion and Shiga toxin production, on bacterial pathogenicity.^59,60^ Only almost two decades later, the HeLa invasion assay led to the initial identification of the T3SS located on the virulence plasmid of the model strain *S. flexneri* 2a required for invasion.^61,62^ The T3SS, a complex bacterial structure that enables the injection of effector proteins directly into the target cells' cytoplasm via specific translocator proteins, has later been identified as a major virulence factor in multiple human, animal and plant pathogens.

Subsequent experiments assigned additional loci on the *Shigella* virulence plasmid to specific virulence events, such as the intracellular escape from the phagocytic vacuole, multiplication in and early killing of host cells.^63–66^ Such highly reductionistic approaches have not only aided the discovery of novel microbial virulence traits and vice versa novel functions of the host cells, but also allowed the detailed dissection of molecular mechanisms of infectious disease processes until today.^67,68^ For example, an RNA interference screen led to the identification of the chemokine receptor CCR5 as a stimulator of pore formation by the T3SS of *Yersinia pseudotuberculosis*.^69^

Upon uptake into macrophages, *Shigella* causes rapid pyroptotic cell death after escape from the phagocytic vacuole by activation of inflammasomes and T3SS effector ubiquitin ligases, which cause degradation of human antimicrobial guanylate-binding proteins.^70,71^ The battery of virulence factors common to all phylogenetically distinct *Shigella* lines has been recently summarized.^72^ Seminal *in vitro* work revealed that intra- and intercellular movement of *Shigella* is determined by actin polymerization via N-WASP on IcsA, a multifunctional type V secretion system autotransporter also involved in cell adhesion, escape from autophagy and extracellular biofilm formation.^73,74^ The IcsA autotransporter delicately interacts with the T3SS to promote virulence and immune escape.^75^ Upon reactivation of the T3SS, the translocon pore protein IcaC interacts with the adherens junction protein β-catenin to reduce membrane tension and promote the formation of cellular protrusions, which are engulfed by adjacent cells resulting in intercellular spread.^76^ While rupturing of the double membrane by *Listeria monocytogenes*, which similarly spreads using the cellular actin polymerization network, is achieved by the pore-forming toxin LLO and phosphatidylinositide phospholipase C, the role of the T3SS effectors IcsB and VirA in the vacuolar escape of *Shigella* needs to be further clarified.^77^

### 3.2 *Salmonella enterica* serovar Typhimurium

Upon contact with animals or ingestion of contaminated produce and animal products, non-typhoidal *Salmonella* serovars, such as *S*. Typhimurium and *Salmonella enterica* serovar Enteritidis, cause a self-limiting gastroenteritis/colitis in immunocompetent individuals characterized by massive influx of neutrophils. In young children and immunocompromised individuals however, invasive disease can manifest, characterized by systemic infection with bacteremia, meningitis and osteomyelitis.^78,79^ In sub-Saharan Africa, such cases have recently seen a dramatic increase in frequency driven by pseudogene formation and chromosomal alterations followed by clonal replacements, resulting in the emergence of the *S*. Typhimurium ST313 clone.^80,81^

*S*. Typhimurium infections are characterized by a massive proliferation of the bacteria in the intestinal lumen. To fuel this overgrowth *S*. Typhimurium has developed chemotactic mechanisms that aid in identifying intestinal niches rich in microbiota- and host-derived electron acceptors, such as the microbial fermentation product 1,2-propanediol.^82,83^ Furthermore, nutrients and metabolites released from dying host cells induce a specific transcriptional response in *Salmonella* that involves expression of the pyruvate-formate lyase, resulting in the promotion of bacterial growth.^84^ While the majority of bacterial cells reside in the gut lumen, few *S*. Typhimurium cells penetrate the mucus and breach the epithelial lining mainly via T3SS-1-mediated invasion of enterocytes. Monolayer culture systems have proven particularly useful in this context due to their compatibility with high-resolution time lapse microscopy, which allowed to directly image pathogen-host interactions at the epithelial cell lining.^85^ A second type III secretion system (T3SS-2) is required for apical-to-basolateral migration and cell egress.^86^ Upon escape from the *Salmonella* containing vacuole, bacteria are marked for autophagy by ubiquitinoylation of their lipopolysaccharide coat by the ring finger protein 213 E3 ubiquitin ligase.^87^

Major virulence determinants of *S*. Typhimurium, adhesion to and invasion of enterocytes and flagellin-provoked induction of a proinflammatory cytokine response in enterocytes can be emulated *in vitro* using cell lines. Seminal studies have resulted in the identification of the T3SS-1,^88^ as well as of fimbriae that mediate adhesion to specific cell types.^89^ Infection of polarized epithelial cells furthermore led to the discovery that flagellin, the subunit of flagella, was rapidly transcytosed, which subsequently stimulated the basolateral secretion of proinflammatory cytokines and the recruitment of polymorphonuclear lymphocytes into the gut lumen.^90^ More recently, dual RNA sequencing of bacteria and human cells (HeLa) upon infection facilitated the profiling of the transcriptomic alterations during the infection process, which was followed up by live cell fluorescent imaging combined with pharmacological interference in both microbes and the host.^67^ The results reveal a novel bacterial small RNA that controls the expression of virulence genes as well as of coding and noncoding host transcripts, thus demonstrating that even simple cellular paradigms remain powerful tools for the dissection of fundamental infectious disease mechanisms.

After infiltration of the intestinal epithelium by few bacterial cells in the context of invasive disease, *S*. Typhimurium can survive in macrophages, which transport microbes to inner organs and are a reservoir for bacteria during chronic subclinical infections.^91^ BMDMs showed a heterogeneity with respect to the response to infection depending on the bacterial status.^92^ Specifically, macrophages display an M1-like pro-inflammatory phenotype with non-growing *Salmonella*, whereas macrophages harboring growing bacteria have an anti-inflammatory phenotype. Within a single BMDM cell, heterogeneity of bacterial replication and cyclic di-GMP second messenger concentration has been observed with a green fluorescent protein dilution and a FRET-based receptor binding assay, respectively, using confocal microscopy. A slow growing population is most affected by (over)production of the exopolysaccharide cellulose, which aids persistence of the organism by virulence downregulation.^93,94^ Activated by the regulator CsgD, the ancient rdar (red, dry and rough colony morphotype upon agar plate growth) biofilm and cyclic di-GMP signaling thus trade off acute virulence versus environmental and host persistence of *S*. Typhimurium.^44,95^ Although not traced back to a specific biofilm phenotype, *in vitro* findings in macrophages suggest that the switch into a transient non-proliferative state allows *Salmonella* to persist during environmental stress such as treatment with antibiotics and reemerge once antibiotic pressure is relieved with important implications for the understanding of recrudescent infection and the prevention of disease relapse.^92,96^

### 3.3 Salmonella enterica serovar Typhi

**Figure 2.**
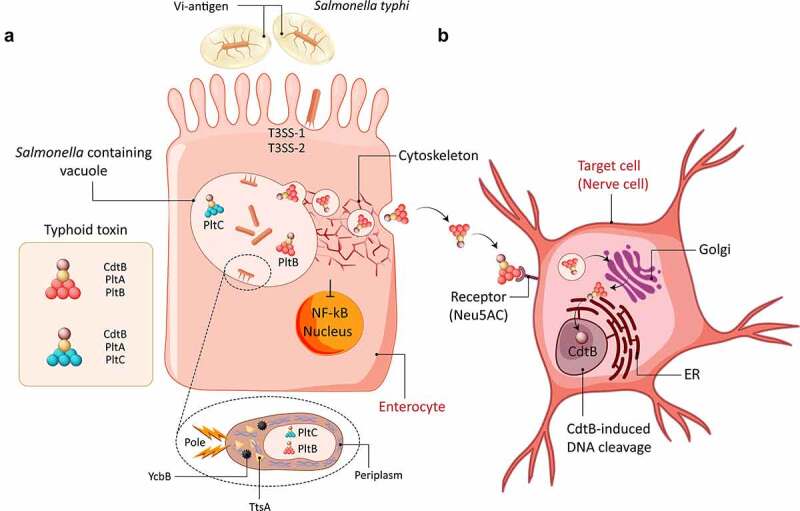
**Schematic overview of typhoid toxin secretion and export, and intracellular trafficking of the typhoid toxin. A)** Typhoid toxin expression commences upon invasion of *S*. Typhi into host cells. Specifically, typhoid toxin consists of two enzymatic subunits, PltA and CdtB, which bind to pentamers of PltB or PltC. Upon assembly, the toxin subunits are secreted into the bacterial periplasm (inset). The bacterial transpeptidase YcbB and muramidase TtsA are required for typhoid toxin secretion into the lumen of the *Salmonella* containing vacuole. Subsequently, typhoid toxin is packaged into vesicle carrier intermediates, which transport the toxin to the plasma membrane where it gets released into the extracellular space. **B)** The fully assembled PltB toxin binds to the *N*-acetylneuraminic acid cell surface receptor (Neu5Ac) which results in the endocytosis and retrograde trafficking to the Golgi complex and endoplasmic reticulum (ER) where the CdtB component is released from its pentameric structure and proceeds into the nucleus to induce DNA damage via its DNase I activity. T3SS = type III secretion system.

As *S*. Typhimurium and *S*. Enteritidis, *Salmonella* Typhi is a distinct pathovar of the species *Salmonella enterica* but, in contrast to the former, is restricted to humans.^97^ *S*. Typhi elicits a severe systemic disease phenotype involving the lymphatic tissue of the gastrointestinal tract and internal organs. Unique virulence factors of *S*. Typhi determinative for the disease phenotype include the virulence-promoting Vi antigen capsule, a linear polymer consisting of α-1,4-linked N-acetylgalactosaminuronate, as well as the typhoid toxin (Figure 2). *In vitro* studies showed that the Vi antigen capsule inhibits phagocytosis and limits the induction of proinflammatory cytokines by epithelial cells and resting macrophages.^98–100^ Typhoid toxin is a composite A_2_B_5_ toxin that is only produced by *S*. Typhi when residing in the *Salmonella* containing vacuoles in host cells. The toxin is a modular assembly of two catalytic A units with distinct catalytic activity, CtdB and PltA, and a pentamer of two alternative B units (PltB or PltC). Although both toxin variants are synthesized, the heteromeric toxin supported by the PltB scaffold is selectively sorted and exported from the vacuole into the extracellular space via vesicles.^68^ Using monolayer cultures of enterocytes, vascular endothelial cells and immune cells, it was shown that the typhoid toxin engages different receptors on different target cell types, including the CD34 family member podocalyxin-like protein 1 (PODXL) on the apical side of epithelial cells, the glycan receptor N-acetylneuraminic acid (Neu5Ac) and the receptor-type phosphatase CD45 expressed on macrophages.^101^ The CdtB catalytic subunit, which exerts DNase I activity subsequently translocates into the nucleus to induce single-strand DNA breaks, triggering DNA damage response and cell cycle arrest.^102^

### 3.4 Vibrio cholerae

Cholera, watery diarrhea caused by distinct strains of the marine and freshwater environmental species *Vibrio cholerae*, is a non-invasive disease of the small intestine. Major virulence factors of the O1 serogroup strains that caused seven pandemics since 1817 are CTX phage derived AB_5_ cholera toxin and the toxin-coregulated pilus TCP located on the vibrio pathogenicity island-1. While the six previous pandemics have been caused by the classical biotype, the seventh ongoing pandemic is caused by the El Tor biotype that differs in virulence factor expression.^103^ After chemotaxis towards the intestinal mucus, *V. cholerae* penetrates the mucus layer via flagellar motility and adheres to enterocytes where it forms biofilms aided by the TCP pilus and other adhesins.^104–107^ The mucin layer is, however, not only a barrier, but directly contributes to the population expansion of *V. cholerae* under aerobic conditions.^108^ Further emphasizing the delicate molecular crosstalk between microbial physiology and the host, microbial proteins such as the recombinant TcpA subunit of the TCP pilus upregulate transcription and production of mucin from 2D cultured HT29 enterocytes and, in turn, mucin exerts a feedback on the expression of the bacterial chitin-binding protein GbpA.^109,110^ Recombinant TcpA further significantly upregulates transcription of Toll-like pattern-recognition receptors (TLRs), proinflammatory cytokines in particular TNF-α and the CEACAM1 adhesin in co-cultures of Caco-2 cells with -peripheral blood mononuclear cells.^111^

The immunogenic neuraminidase (sialidase) recruits additional monoganglioside GM1 glycosphingolipid receptors to the host cell plasma membrane of 2D cultured primary human colon cells, which further facilitates uptake of cholera toxin into enterocytes.^112,113^ Alternatively, cholera toxin can also be delivered to intestinal epithelial cells in outer membrane vesicles that protect it from intestinal proteases.^114^ Upon entry, the catalytic subunit of cholera toxin ADP-ribosylates the α subunit of GTP-binding proteins (G_s_) to irreversibly inhibit its GTPase activity, resulting in constitutive activation of adenylate cyclase. Excessive cyclic AMP concentrations then activate protein kinase A (PKA), which phosphorylates its target, the chloride channel CFTR, leading to Cl^−^, HCO3^−^ and water efflux.^115^ Na^+^ counterions are recruited via the paracellular route which cumulatively leads to massive dehydration, thus directly linking the molecular mechanisms dissected *in vitro* to the observed disease symptoms. Besides this mechanism cholera toxin exerts additional functions such as targeting Rab11, the major regulatory hub of intracellular membrane trafficking, via cyclic AMP which leads to inhibition of exocyst trafficking and disruption of barrier integrity.^116^

### 3.5 Pathogenic *Escherichia coli* strains

The role of the species *Escherichia coli* in interaction with humans is multifactorial.^117^ *E. coli* can even be considered a symbiont, as it is among the first species colonizing the gastrointestinal tract, which stimulates the innate immune response and significantly protects against the development of autoimmunity.^118^ However, with a tenuous transition to a pathogenic lifestyle via acquisition of accessory genomic islands bearing distinct colonization and virulence factors, *E. coli* has diversified into numerous intra- and extra-intestinal pathovars.^119^ Conventional Caco-2 monolayer cultures have had notable success in the development of anti-infectives that act via blocking the bacterial machinery for enterocyte entry. Specifically, the pharmacological inhibition of the bacterial Tir protein that upon injection into enterocytes remodels the cytoskeleton to provide susceptibility to enteropathogenic *E. coli* (EPEC) infection, blocked infection *in vitro* and protected mice from EPEC-induced diarrhea.^120^

*E. coli* is also associated with the development of chronic inflammatory diseases, such as Crohn’s disease, and with the development of gastrointestinal cancer. Inflammatory disease manifestations are associated with the adherent-invasive pathovar that displays distinct biological features, including the formation of intracellular colonies in immune cells.^121^ In contrast, development of colorectal cancer is associated with specific *E. coli* strains that encode genotoxins, such as colibactin, which can alkylate DNA in cell culture and *in vivo* directly leading to genomic instability.^122,123^

### 3.6 Campylobacter jejuni

The helically shaped polarly flagellated Gram-negative bacterium *Campylobacter jejuni* is the most prevalent food-born pathogen with one in ten individuals globally affected each year. Colonizing warm-blooded animals including birds, the organism is transmitted by consumption of undercooked poultry, but also milk and water. *C. jejuni* strains have been found to cause disease with a broad range of symptoms from watery to bloody mucoid dysentery. Additionally, infections can be followed by a number of autoimmune diseases, such as Guillain-Barré syndrome. Although the disease symptoms had been recognized already by Theodor Escherich in the 19^th^ century, *Campylobacter* was isolated only in the 1970ʹs.^124^ Molecularly, the periplasmic protease HtrA is the virulence factor that enables rapid paracellular translocation of *C. jejuni* by cleavage of the adherens and tight junction proteins, such as E-cadherin and occludin, without however altering overall membrane integrity, as evidenced by unchanged transepithelial resistance in polarized epithelial transwell cultures upon infection.^125^

Adhesion of *C. jejuni* to epithelial cells is in part mediated by interactions between host heparin and the bacterial flagellar tip protein FliD, as well as by direct, high-affinity interactions of the O-side chain of *Campylobacter* lipooligosaccharide with host glycans.^126,127^ Following binding, the fibronectin-binding protein CadF activates the integrin-FAK signaling axis to trigger actin rearrangement and bacterial invasion.^128^ Using an infection model based on co-cultured human enterocytes and HT29-MTX mucin-producing goblet-like cells in transwells, *Campylobacter* adhesion and invasion was shown to require N-linked glycosylation of *C. jejuni* proteins and a unique O-methyl phosphoramidate modification of its capsular polysaccharide.^129,130^

Interestingly, *C. jejuni* lacks a T3SS that is common to most enteropathogenic bacterial species. In its absence, cytotoxicity to different human cell culture models, including HeLa and Caco-2, is exerted by a variety of factors, including functional flagella, secretion of cytolethal distending toxin (CDT), as well as membranous short-chain lysophosphatidylethanolamine that permeabilize host cells.^131,132^ Another unconventional virulence factor is the CRISPR/Cas related endonuclease CjeCas9 that causes unspecific DNA cleavage and programmed cell death upon its translocation into the nucleus.^133^ Furthermore, the *Campylobacter* metabolite ADP-heptose, a core oligosaccharide precursor of lipopolysaccharide, strongly activated NFκB signaling in enterocytes, without the need for a type III or type IV injection machinery.^134^ Similar effects have been observed in other pathogens, including *Helicobacter pylori* and *Akkermansia muciniphila*, suggesting that ADP-heptoses constitute a pathogen-associated molecular pattern.^135–137^

## 4. Advanced intestinal culture systems

As illustrated above, 2D monolayer cultures of human intestinal cells – either as enterocyte monocultures or co-cultured with goblet cells or immune cells – have contributed, and continue to contribute, tremendously to our mechanistic understanding of the pathogenic processes underlying enteric bacterial infections. Specifically, these culture systems have proven very useful for 1) the systematic identification of host factors required for or protective against infection, 2) the determination of virulence factors and their regulatory pathways, as well as 3) the uncovering of metabolites and post-translational modifications that link infection to cellular pathogenesis. To these ends, RNA interference, library screens based on CRISPR/Cas or transposons and dual RNA sequencing complement previously used fluorescence microscopy, pharmacological interference and biochemical strategies such as systematic mapping of protein-protein interactions for the detailed dissection of host regulatory pathways.

Notably however, despite these undisputable technological advancements, monolayer cultures are not well suited for discovery studies that require long-term maintenance, accurate cellular and molecular phenotypes (e.g. to support complex pathogen lifecycles or to mimic changes in infectivity along the gastrointestinal tract), crosstalk between different enteric cell types, peristaltic motion or interactions between the pathogen and a complex commensal microbiome. Furthermore, studies using cell lines are not well positioned to investigate effects of inter-individual differences. To overcome these limitations, a wide variety of diverse organotypic and microphysiological *in vitro* and *ex vivo* models have been developed in recent years. Importantly, we would like to emphasize that we see these more complex systems not as a replacement for conventional monolayer studies, but rather as a useful complement to address selected aspects that cannot be interrogated in the more simplified culture systems.

### 4.1 Intestinal organoid culture

Advances in stem cell research have facilitated the development of 3D cell culture systems that aim to narrow the gap between 2D monotypic cultures and *ex vivo* tissue models. Specifically, intestinal organoids have provided an important breakthrough for the emulation of intestinal phenotypes and functions.^138,139^ Intestinal organoids can be derived from tissue-resident adult stem cells, embryonic stem cells (ESCs) or induced pluripotent stem cells (iPSCs) using Matrigel as a scaffold. In culture medium containing specific mitogens and differentiation factors, these cells proliferate and, following a series of divisions, self-organize into microtissues containing enterocytes, as well as goblet cells, Paneth cells and enteroendocrine cells.^140^ Intestinal stem cells can furthermore be induced to differentiate into M cells by exposure to the TNF family cytokine RANKL.^141^ Organoids exhibit the morphology of the folded intestinal epithelium including villi and crypt-like domains the latter of which host a functional stem cell niche at their base.

In recent years, organoids have been adopted for modeling infection with pathogens. These systems hold promise for addressing novel questions in host-microbe interactions, infectious diseases, and the resulting inflammatory conditions. As organoids capture the cellular complexity of the human intestinal epithelium, they are well suited to investigate pathogens with narrow cell-type tropism and the corresponding mechanisms of their cell-specific interactions. For instance, M cells are the preferred cell type for *S. flexneri* invasion and the presence of these cells in the intestinal organoid enabled the holistic analysis of this infection.^142^ Importantly, however, when conventional organoid protocols are used, the apical side faces inwards, which requires that microbiome and pathogen need to be microinjected. Multiple pathogens have already been injected into organoids to understand intestinal disease mechanisms, including enterohemorrhagic *E. coli*,^143^ *Cryptosporidium parvum* ^144^ and *S. enteritica*.^145,146^ Furthermore, organoids can support the luminal culture of microbial communities from human fecal isolates including both aerobic and obligately anaerobic taxa for at least four days.^147^ However, microinjection is labor intensive, and the multiplicity of infection (MOI) is difficult to control given the differing numbers of cells per organoid. While there are elegant technical solutions to inject organoids at scale, these methods require considerable technical expertise as well as investments into robotic infrastructure.^147^ To ameliorate these issues, protocols for the generation of inverted, apical-out organoids have been established.^148^ These methods are based on the removal of extracellular Matrigel and subsequent culture in low attachment plates, resulting in reversed polarity while functionality and barrier integrity stayed intact. These inverted organoids recapitulated the preferential infection with *S*. Typhimurium and *L. monocytogenes* on the apical and basolateral sides, respectively.^149^ However, these methods instead render the basolateral side inaccessible and do not solve issues pertaining to controlling MOI.

To address these limitations, an alternative approach is the monolayer culture of organoid-derived cells. For this approach, organoids are enzymatically dissociated into single cells and then seeded on transwells, thereby ensuring appropriate polarity and barrier formation while retaining the cellular complexity of the intestinal epithelium and granting ready access to both apical and basolateral sides. By generating organoids from patient-derived samples with region-specific development into mature epithelial lineages, this approach has been successfully used for infections with *S. flexneri*, different pathogenic *E. coli* strains as well as with *S*. Typhi and *S*. Typhimurium.^37,150,151^ Specifically, the cultures closely recapitulated *in vivo* tissue tropism, inter-individual differences in infectivity as well as pathogen-specific infection patterns. For *Shigella*, organoid-derived monolayer cultures revealed basolateral invasion preference in the absence of M cells,^152^ whereas in the presence of M cells also direct lateral M cell-to-enterocyte spread can be observed.^57^ Transwell culture of dissociated organoids has moreover identified the infection dynamics and coordinated contractile responses of reconstituted intestinal epithelium upon sensing of the T3SS and flagellar ligands of *S*. Typhimurium.^153,154^ Organoid-derived monolayers also allow to study microbial uptake by M cells, as demonstrated for the enteric pathogen *Yersinia pseudotuberculosis*, which exploits M cells to gain access to the underlying lymphatic system.^155^

The physiologically relevant phenotypes are showcased by the possibility to culture previously non-cultivatable pathogens in monolayer cultures of dissociated organoids, such as norovirus ^156,157^ and rotavirus,^158,159^ which are among the most common causes of gastroenteritis. Furthermore, culture of intact or dissociated organoids on air-liquid interface transwells allowed for the first time to support the entire life cycle of *Cryptosporidium* spp, apicomplexan parasites, which infect enterocytes and propagate via rounds of release and reinvasion with both sexual and asexual replication stages.^144,160^ As such, this culture paradigm is highly promising to enable studies into the molecular biology of meiotic divisions and for progressing genetic analyses of complex apicomplexan phenotypes.

**Figure 3.**
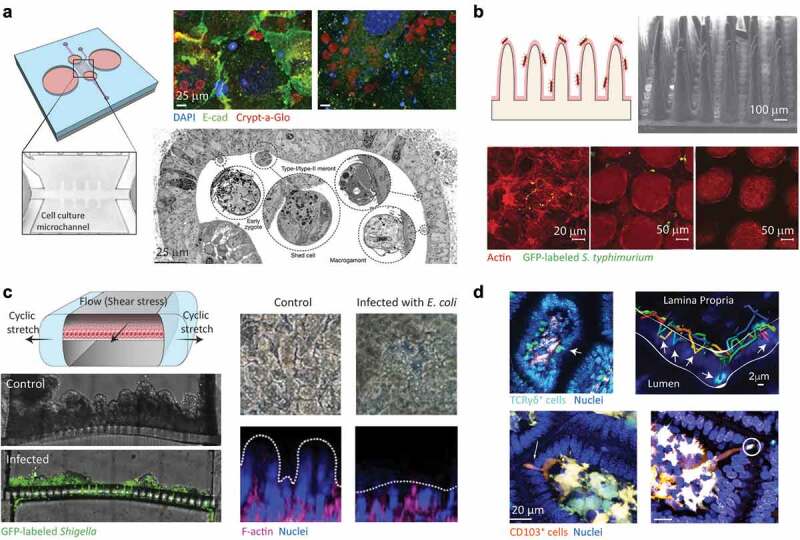
**Examples of organotypic and microphysiological culture methods to mimic enteric infections. A)** Schematic of a fluidic device based on dissociated intestinal organoids. Inset shows the hydrogel-based microchannel. Immunofluorescence of *C. parvum* undergoing its major epicellular stages in the mini-guts (right-top) and scanning electron microscopy image of distinct stages of the *C. parvum* life cycle at 72 h post infection (right-bottom). Figure obtained with permission from.^162^ **B)** Illustration showing a 3D villi model infected with *S*. Typhimurium. Scanning electron microscopy image of the fabricated villi (top right), and fluorescence micrographs showing *S*. Typhimurium in 2D (bottom left), the crypt section of 3D villi (bottom-middle) and the villus tips of 3D villi (bottom right) 20 days after infection. Image obtained with permission from.^163^ **C)** Schematic of a cross-section of an intestinal microphysiological system (left top). Micrographs showing cross-sections of uninfected (control) and chips infected with GFP expressing *Shigella* (bottom left). Phase contrast images, and fluorescence confocal micrographs (vertical cross-sectional views) of villi showing intestinal villus damage upon infection with enteropathogenic *E. coli* (right). Images in this panel were obtained with permission from.^164,165^ **D)** Examples of intravital microscopy (IVM). Top: IVM analyses of TCRγδ^GFP^ reporter mice after infection with *S*. Typhimurium. Arrows show tracked flossing movements on one villus (left) and 4D tracking of TCRγδ^GFP^ cells (right). The intraepithelial compartment is outlined. Reproduced from ^166^ with permission. Bottom: IVM analyses of *Salmonella* uptake by CD103^+^ dendritic cells showing that the immune cells extend dendrites through the epithelium while crawling above the basement membrane (arrow on left); the dendrites engulf *Salmonella* (circled on right), and retract them toward the cell’s soma. Images adopted with permission from.^167^

Intestinal organoids are easy to maintain, can be readily established from individual patients for personalized testing and provide physiologically relevant and versatile tools that closely mimic the human intestinal epithelium. However, notable hurdles and limitations intrinsic to the method remain. It is important to consider that Matrigel, which constitutes the most commonly used scaffold for intestinal organoids, commonly contains gentamicin, which interferes with bacterial cultures.^161^ Furthermore, while organoids accurately recapitulate the composition of the epithelial lining, they lack immune cells, neuronal connections and vascularization, as well as flow and peristaltic motion. Thus, combination of organoid culture and tissue engineering approaches, as demonstrated for the reconstruction of tubular epithelia with an accessible lumen connected to external micropumps (Figure 3A), could be promising to combine cellular complexity with an appropriate biophysical microenvironment, thereby further approximating intestinal architecture and function.

### 4.2 Models on bioengineered scaffolds

With the emergence of tissue engineering in the 1990s, models have been developed in which cells are cultured on specific 3D scaffolds that resemble the structure of the tissue *in situ*. These models are specifically a breakthrough for intestinal studies given the complex native crypt-villus geometry, which is required for efficient intestinal stem cells self-renewal and for the generation of biochemical gradients that mediate cell communication, differentiation and proliferation.^168,169^ A variety of biomaterials have been used to create the scaffolds, ranging from biocompatible hydrogels based on natural or synthetic biopolymers with tunable physiochemical properties ^170,171^ to decellularized extracellular matrix.^172^ Besides the biochemical scaffold composition, also its stiffness impacts the morphology and differentiation of intestinal epithelial cells. Specifically, stiff substrates result in flattening of the cells whereas physiological matrix elasticity results in columnar organization and the maintenance of cellular polarity.^173^ Decellularized scaffolds offer the natural composition and physical properties of the tissue for cells and the intestinal submucosa scaffold is widely used to model intestinal epithelium *in vitro*. Seeded with Caco-2 cells, these scaffolds were used to investigate the infection with *C. jejuni*. It was observed that non-capsulated bacteria can adhere and internalize into the cells in 3D, whereas this effect was not seen in monolayers cultured on flat topologies.^174^ This model was later advanced by the incorporation of endothelial cells and monocytes. When the co-culture system was infected by *S. enterica*, the pathogens were confirmed to target epithelial cells only, while the endothelial cells remained intact.^175^

In addition to these native scaffolds, bioengineered 3D structures are widely used for modeling bacterial infections in order to increase standardization and avoid the dependence on fresh biological specimens. These scaffolds are produced using synthetic polymers such as poly(lactic-co-glycolic acid) (PLGA) ^176,177^ or poly(ethylene)glycol diacrylate (PEGDA) ^178^ which are then coated with hydrogels or ECM derived proteins.^163,171^ Using such an approach, collagen-based 3D villi scaffolds were fabricated and seeded with Caco-2 cells to investigate interactions between the human intestinal epithelium and enteric pathogens (Figure 3B). This reconstitution of the intestinal topology resulted in increased expression of mucins scaffold-cultured cells compared to 2D culture.^163,179^ The mucin layer protected the epithelial cells from infection with the prototypic adherent-invasive *E. coli* strain LF82 or invasive *S*. Typhimurium, which was reversible by knock-down of the mucin MUC17. 3D scaffolds have also been used for the culture of cells derived from dissociated intestinal organoids, resulting in the development of a physiologically relevant mimic of the human small intestinal epithelium with crypt-villus architecture, appropriate cell-lineage compartmentalization and dedicated stem cell niches.^171^ 3D bioengineered scaffolds can furthermore be integrated with the culture of intact organoids. Specifically, a 3D collagen scaffold with physiological topography was presented in which primary intestinal fibroblasts were integrated to mimic the intestinal stroma.^180^ Culture or intestinal organoids on this scaffold resulted in the epithelialization of the scaffold and the correct formation of functionally distinct villi and crypt domains without the need for organoid dissociation, thus providing a versatile means for future infectious disease studies.

An interesting recent development is the bioprinting on 3D scaffolds. Using bioinks containing a decellularized small intestine submucosa, Caco-2 cells and human umbilical vein endothelial cells (HUVECs) were printed to reconstruct an intestinal epithelium with high aspect ratio 3D villi and a connected underlying capillary network.^181,182^ The model closely mimicked the geometry of human intestinal villi, exhibited enhanced barrier function and expressed higher levels of intestinal enzymes (ALP and ANPEP) and mucins (MUC17), markers of mature enterocytes, compared to either conventional monolayers or 3D models without endothelial bed. While such bioprinted systems are promising to further increase the level of detail at which human intestinal physiology can be modeled, they have not yet been utilized for the study of enteric infections. Furthermore, lack of flow, shear and peristalsis, as well as the impedance of diffusion caused by the polymeric matrix remain important to consider for studies based on scaffold-based 3D models.

### 4.3 Microfluidic devices (“Gut-on-a-chip”)

Advances in microengineering technologies have resulted in the development of microphysiological systems in which cells are cultured in a perfused environment with hydrostatic forces and mechanical deformations. Arguably the first perfused intestinal model was based on a system comprising two microchannels which were divided by a rigid semipermeable membrane on which Caco-2 cells were seeded.^183^ The model presented an important step forward in the development of biomimetic *in vitro* models, but it still lacked relevant physiological features such as villi structures, cellular complexity and peristaltic motions. To address these limitations, the rigid polyester membrane was replaced with a porous flexible polycarbonate membrane and two vacuum chambers were integrated on the sides of the device to introduce cyclic strain and emulate peristaltic motion.^184^ Application of microfluidic flow and mechanical strain resulted in improved enterocytic phenotypes, mucus production, metabolic activity and the formation of crypt-villi microstructures with subsets of cells differentiating into Goblet and enteroendocrine cells.^185,186^ Upon infection with *S. flexneri*, bacteria rapidly colonized the crypt-like invaginations resulting in high rates of infectivity with minimal bacterial loads in the microphysiological system, which were further amplified by peristaltic forces (Figure 3C). In contrast, infectivity in monolayer culture was 10,000-fold lower.^164^

Model complexity was further increased by co-culture with endothelial cells at the opposite side of the membrane. Strikingly, exposure of such co-cultures to bacterial lipopolysaccharides on the endothelial (basolateral) side resulted in increased production of pro-inflammatory cytokines and loss of barrier integrity, whereas exposure on the apical side did not result in inflammation, but even further increased epithelial tightness.^187^ Such selective immunotolerance is critical for meaningful studies of host-microbe interactions. The relevance and potential of these systems was furthermore demonstrated by co-cultures with commensal microbes. Specifically, colonization of the microphysiological model with a probiotic formulation of eight strains of beneficial bacteria (VSL#3 formulation) resulted in more *in vivo*-like transcriptomic profiles, as well as inhibition of pathogenic *E. coli* growth and resulting epithelial injury.^165^ Importantly, microfluidic models are moreover compatible with the stable co-culture of communities of aerobic and anaerobic human gut microbiota and application of a physiological oxygen gradient in an engineered anaerobic chamber allowed to sustain relevant levels of microbial diversity, consisting of over 200 unique operational taxonomic units from 11 genera.^188^

These results demonstrate that the continuous or frequent-pulse flow in microfluidic systems that replenishes nutrients and removes metabolic waste products as well as non-adherent bacteria, prevents bacterial overgrowth and extends model stability from a few hours to multiple days, thereby providing powerful tools for the emulation of microbial homeostasis and imbalance. Combined, these engineered models allow to combine 3D intestinal architecture, biochemical gradients, mechanical cues and microbial complexity to stimulate the organization and differentiation of primary cells. These models thus provide the most biomimetic *in vitro* models for bacterial infection available to date.

### 4.4 *Enteric* ex vivo *models*

*In vitro* assays constitute reductionistic approaches that allow to probe and functionally challenge intestinal biology and pathobiology in accessible systems. It is important to note though that these technologies are reductionistic and even the most advanced culture systems do not include the diverse populations of innate and adaptive immune cells present in the human mucosa and cannot mimic innervation, vascularization or muscular coating. For this reason, *ex vivo* cultures, ranging from the short-term culture of intestinal slices or explants to intravital analyses of intestinal loops in anesthetized animals, constitute important complements to the arsenal of intestinal *in vitro* systems. Specifically, ileal loops have served as an important model for the study of enteric bacterial infections for many decades. In this paradigm, animals, mostly rabbits or mice, are laparotomized, followed by the exposure of a few centimeters of intestinal loops, which are carefully ligated and injected with a defined bacterial suspension. Subsequent histological and biochemical evaluation of the infected tissues and non-infected control loops have revealed important disease mechanisms and host responses and have enabled the development of protective strategies against various enteric infections, including *S. flexneri, Clostridium perfringens*, enteropathogenic *E. coli* and *C. jejuni*.^189–193^

The arguably most common *ex vivo* culture model is based on the Ussing chamber system, an experimental apparatus in which an epithelium, e.g. obtained from an intestinal biopsy, is mounted vertically to separate two chambers. By measuring electric potential differences between the electrodes in the adjacent compartments, the setup can quantify epithelial barrier properties, permeability as well as ion transport. Ussing chambers have been used to study the effects of various enteric pathogens on intestinal integrity. For *S. flexneri* infections, Ussing chamber experiments have facilitated the identification of enterotoxins and demonstrated their rapid and dose-dependent effects of intestinal absorption.^194,195^ Moreover, enterohemorrhagic but not non-pathogenic *E. coli* was identified to increase expression of the receptor for the neuropeptide galanin 1 and subsequent exposure to galanin increased short-circuit currents in the Ussing chamber by 20-fold, linking the molecular pathogenesis to the excessive fluid secretion in bacterial dysentery.^196^ Similar secretory responses with disruption of intestinal integrity were found for *C. jejuni*,^197^ *Clostridioides difficile* ^198^ and *Enterococcus faecalis*.^199^ Furthermore, *ex vivo* infection of mounted human ileal biopsies with *S*. Typhi followed by transcriptomic profiling revealed that the host mucosal immune response was downregulated already during the first two hours of invasion.^200^

Cultures of precision-cut slices from mice, rat, chicken and human intestines have emerged as model systems for the evaluation of intestinal metabolism and toxicity.^201^ These systems accurately reflect the cellular and structural complexity of the tissue *in situ* with myenteric and submucosal neuronal plexuses and functional interstitial cells of Cajal.^202^ However, their functional stability is restricted to a few hours and, as such, these systems are currently not in widespread use for studies of enteric infections.

Intravital microscopy (IVM) of intestinal loops plays a significant role in elucidating the host immune response of macrophages, dendritic cells, T cells, B cells, and various innate-like lymphocytes to enteric infections.^203^ These methods draw on the extensive repertoire of fluorescent imaging methods to study intestinal barrier transport of pharmacologically active peptides.^204^ Upon infection with *S*. Typhimurium, multiphoton imaging of these loops showed that intraepithelial lymphocytes augmented scanning behavior by increasing their motility and squeezing in between the intestinal cells.^166^ Furthermore, dendritic cells concentrated in the epithelium and efficiently phagocytose bacteria by pulling them from the lumen using intraepithelial dendrites.^167^ Similarly, CX3CR1^+^ cells were observed to rapidly migrate into the intestinal lumen in the vicinity of the *Salmonella* clusters and control the initial infection ^205^. Notably, peristalsis and the resulting movement of the tissue challenges the acquisition of stable images and, thus, advanced software tools are required to correct and improve the visualization of dynamic events, such as cell-cell interactions.^205^

## Open questions

As outlined above, intestinal *in vitro* and *ex vivo* models have contributed extensively to our understanding of the pathobiology of enteric diseases. However, multiple important frontiers remain. Although pathogen-host interactions have been studied in great detail, much less is known about the roles of the microbiota in colonization resistance, but also in promotion of infections upon dysbiosis. Recently developed models which allow for more long-term concomitant exposures to microbiome components and pathogens are starting to shed light on these intra-kingdom interactions ^206–208^ and we anticipate that these and other investigations will provide important experimental insights into the interplay between the microbial flora and intestinal health. Along similar lines, unravelling of the molecular mechanisms underlying the beneficial roles of probiotics on the human host beyond the simple prevention of pathogen exclusion can contribute to new insights into human well-being.

Another important aspect that we find to be currently understudied is the impact of genetic and epigenetic factors in both microbes and host on infectivity and pathogenesis. Polymorphisms in multiple human immune-related genes, including various interleukins and genes encoding the major histocompatibility complex, have been implicated in the susceptibility to enteric infections.^209^ However, these genetic association studies are often underpowered, lack replication in independent cohorts and are not supported by functional assays, which is of particular importance for synonymous variations or variants in non-coding regions of the genome. We believe that organoid culture that is amenable to genetic editing provides an important opportunity to directly probe the effects of genetic variations on infectious disease phenotypes. Similarly, *in vitro* systems allow for the systematic investigations of genetic variability at the level of the pathogen.

## Concluding remarks

**Figure 4.**
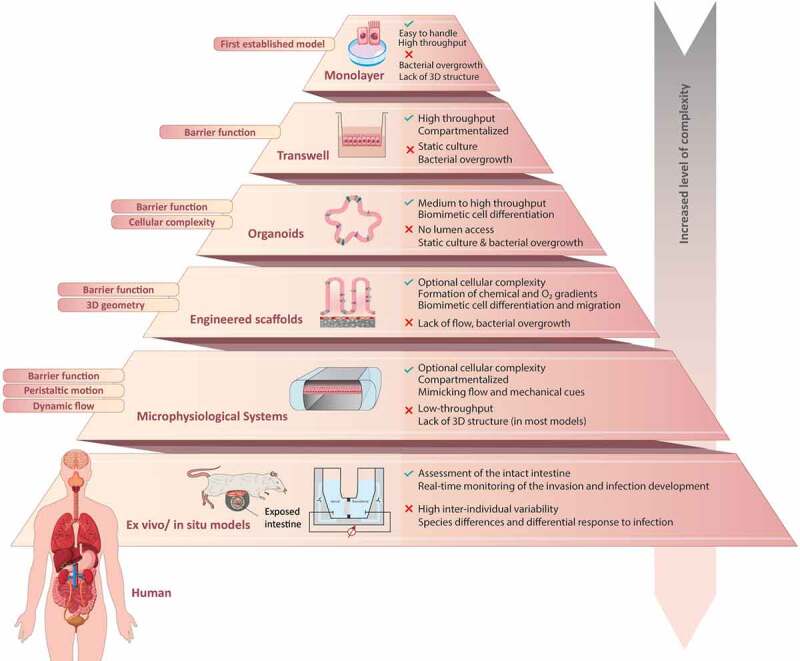
**Representative schematic of biological models applicable to study enteric bacterial infections and host-pathogen interactions in the intestine**. The specific features, as well as advantages and limitations of intestinal model systems are highlighted and compared with respect to complexity, physiological relevance and scalability. Note that transwells, engineered scaffolds and microphysiological systems are compatible with the culture of cell lines, primary intestinal cells as well as dissociated organoids^211^.

Enteric infections remain a main global health concern accounting for >1.6 million deaths per year, more than 25% of which in children under the age of five.^210^ While minimizing malnutrition, contaminated drinking water and unsafe sanitation constitute the main means for the prevention of severe disease, obtaining a deeper mechanistic understanding of gut homeostasis and pathogenesis is of paramount importance for the development of new therapies. Experimental model systems that faithfully reflect infectious disease biology constitute essential tools in this regard. Over the past 60 years, conventional 2D monolayer cultures have played critical roles in the identification of factors in both microbes and host cells that are critical for infectivity. More recently, this culture method has been complemented with a multitude of 3D *in vitro* and *ex vivo* systems that differ in complexity, stability, versatility and function (Figure 4).

Monolayer cultures remain the method of choice for high-throughput applications, such as large-scale screening based on transposon mutagenesis, RNA interference, genetic editing or pharmacological modulation. Intestinal organoids provide unprecedented opportunities to reconstruct the cellular complexity of the human intestine *in vitro* and they provide critical building blocks for bioengineered systems. Major advantages of 3D scaffolds and microphysiological systems are the ability to reconstitute intestinal tissue architecture and integrate biochemical gradients, flow and mechanical cues, which extends the functional lifespan of *in vitro* cultures, allows the cultivation of previously non-cultivatable pathogens and facilitates the incorporation of a physiologically relevant and stable microbiome. Lastly, *ex vivo* cultures of intact tissue explants and intravital imaging of ligatured intestinal loops have facilitated discoveries into the effects of enteric infections on fluid secretion, as well as into the behavior of immune cells upon infection in their native microenvironment. Thus, instead of being replacements for conventional 2D cultures, we see these emerging, complex *in vitro* and *ex vivo* systems as complements that each allow to address unique sets of questions.
